# The Role of Presepsin in Diagnosing Infections in Patients with Liver Cirrhosis and Overt Hepatic Encephalopathy

**DOI:** 10.3390/diagnostics12092077

**Published:** 2022-08-27

**Authors:** Razvan Igna, Irina Gîrleanu, Camelia Cojocariu, Cristina Muzîca, Laura Huiban, Catalin Sfarti, Tudor Cuciureanu, Stefan Chiriac, Ana-Maria Sîngeap, Oana Cristina Petrea, Remus Stafie, Sebastian Zenovia, Robert Năstasă, Ermina Stratina, Adrian Rotaru, Carol Stanciu, Anca Trifan, Mihaela Blaj

**Affiliations:** 1 Department of Gastroenterology, Grigore T. Popa University of Medicine and Pharmacy, 700111 Iasi, Romania; 2 Intensive Care Unit, “St. Spiridon” University Hospital, 700115 Iasi, Romania; 3 Institute of Gastroenterology and Hepatology, “St. Spiridon” University Hospital, 700115 Iasi, Romania

**Keywords:** presepsin, liver cirrhosis, infections, hepatic encephalopathy

## Abstract

Infections and sepsis represent severe liver cirrhosis (LC) complications and the precipitating factors of hepatic encephalopathy (HE). The early diagnosis and treatment of infections in patients with LC and HE can significantly increase their survival. Presepsin is a serum biomarker evaluated for the early diagnosis of infections and sepsis in the general and cirrhotic populations. This study aimed to evaluate the role of presepsin in the early diagnosis of infections in patients with LC and HE. This prospective observational study included all consecutive cirrhotic patients admitted to our tertiary university center with overt HE. The patients were follow-up until discharge. In this study, we included 365 patients with a median age of 59 years, of whom 61.9% were male. Infections were diagnosed in 134 patients (36.7%). The presepsin level was higher in patients with infections than those without infections (3167 vs. 500, *p* < 0.001). The ROC analysis results demonstrated that the best cut-off value for presepsin in infections detection was 980 pg/mL with a sensitivity of 80.17%, specificity of 82.5% (AUROC 0.869, CI 95%: 0.819–0.909, *p* < 0.001, Youden index J of 0.622), a positive predictive value of 40.63%, and a negative predictive value of 96.53%. In conclusion, in patients with LC and overt HE, presepsin levels >980 pg/mL could enhance the suspicion of bacterial infections. Presepsin may be an adequate non-invasive tool for the early diagnosis of infections in patients with LC and overt HE.

## 1. Introduction

Liver cirrhosis (LC) and its complications are among the leading causes of mortality worldwide, reaching 30% per month and 63 % per year [1,2]. Bacterial infections are the precipitating factors of LC decompensation, acute-on-chronic liver failure development, and hepatic encephalopathy (HE) [2,3]. The early diagnosis and treatment of infections and sepsis in LC are associated with increased patient survival. Postponing the antibiotherapy initiation for as long as one hour increases the mortality rate by 7.6% [3,4,5].

HE is a complication of LC precipitated by upper digestive bleeding, dyselectrolitemia, constipation, or infections with or without sepsis [2]. The early diagnosis of infections is challenging in patients with LC and HE as alert mental status is a clinical sign of HE and diagnostic criteria for sepsis [6]. Moreover, leucopenia, arterial hypotension, or hypoxia could represent a consequence of portal hypertension and not the signs of infection. Considering all these data, an early diagnosis of infections is difficult in LC, especially in patients with overt HE. In this case, serum biomarkers should be identified for an early infection diagnosis.

Different biological markers were tested over time to diagnose infections and/or sepsis in patients with LC [7,8,9,10,11]. C-reactive protein (CRP), procalcitonin (PCT), and presepsin are practical to be used in daily clinical practice and were evaluated in patients with different stages of LC as the markers of infections or sepsis [10,11,12]. It should be noted that CRP is an acute phase reactant synthesized by the liver, thereby influenced by LC severity [13], and procalcitonin has a renal clearance and is affected by acute or chronic kidney injury [14,15].

Presepsin is one of the biological markers efficient in the early diagnosis of infections in LC [11]. Presepsin is a biological marker produced by the cleavage of soluble CD14. CD14 is a co-receptor for bacterial ligands, including the lipopolysaccharide (LPS) complex of gram-negative bacteria (GNB), making presepsin a biomarker of innate immune activation [9,14,16]. Presepsin can recognize gram-negative and gram-positive bacteria and represent the activation of monocytes in contact with bacterial pathogens; however, it is not synthesized by the liver [14,17].

In the general population, presepsin was an acceptable marker for diagnosing sepsis and systemic infections [16]. A few studies have evaluated the diagnostic value of presepsin in infection diagnosis in patients with LC. Some concluded that presepsin is an acceptable biological marker for the diagnosis of infections in LC [9,11,18] and that increased presepsin level could be associated with a poor prognosis regarding the Model of End-Stage Liver Disease (MELD) score [11,19]. Some other studies documented that PCT and CRP are more appropriate markers for bacterial infection detection in patients with decompensated LC [10,19]. However, no study has assessed the relationship between presepsin and infection diagnosis in patients with LC and overt HE.

This study aimed to evaluate the role of presepsin in the early diagnosis of bacterial infections in patients with LC and overt HE.

## 2. Materials and Methods

### 2.1. Patients

This prospective observational study included all consecutive adult patients with liver cirrhosis and overt HE admitted to our tertiary university hospital from 1 January 2020 to 31 December 2020. The patients were follow-up during hospitalization.

The following patients were excluded from the study: individuals without HE and those with covert HE, patients receiving recent (less than one week) antibiotic treatment, excepting rifaximin, prior admission, patients with severe pulmonary or cardiac comorbidities, those with end-stage malignancies, including hepatocellular carcinoma, individuals with fungal infections, and those with nosocomial infectious complications.

The study was approved by the Local Ethics Committee (No. 102/10.10.2019). The study was conducted according to the principles of the Declaration of Helsinki. All patients or legal representatives signed informed consent before entering the study.

### 2.2. Clinical and Laboratory Assessment

Liver cirrhosis was diagnosed according to the clinical, laboratory, and imaging data. All patients were in a decompensated stage of the disease. The severity of LC was assessed using the Child-Pugh class and MELD scores. HE was graded according to the West Haven criteria [20,21].

The bacterial infections were diagnosed based on clinical manifestations, physical examination, and biological and imaging tests. All patients were screened for infection at admission.

Spontaneous bacterial peritonitis was diagnosed if the absolute polymorphonuclear leukocyte count in the ascites was >250 cells/mm^3^ or if the ascitic fluid cultures were positive [22]. Urinary tract infection would be considered if the urine culture had >10^5^/mL bacterial colony counts. Bloodstream infections would be diagnosed if the patient had positive blood culture. *Clostridioides difficile* infection (CDI) was diagnosed by the presence of stool A and/or B *Clostridioides difficile* toxins in patients with watery diarrhea. Pneumonia was diagnosed based on symptoms and suggestive chest X-ray images. The skin and soft tissue infections were diagnosed according to clinical examination and positive cultures.

The infections were considered healthcare-associated if the patient had a history of hospital admission less than eight weeks before hospitalization. The rest of the infections were community-acquired [23].

The serum presepsin level was measured on admission using the chemiluminescent enzyme immunoassay method and routine laboratory tests (liver and renal biochemistry, complete blood count, INR, CRP, white blood cell count, neutrophil-to-lymphocyte ratio- NLR). For presepsin determination, we used a PATHFAST^®^ presepsin analyzer (Mitsubishi Chemical Medience Corporation, Tokyo, Japan). The method’s detection limit was 20 pg/mL.

### 2.3. Statistical Analysis

The data were analyzed with SPSS software version 20.0 (SPSS Inc., Chicago, IL, USA). Categorical variables were expressed as percentages and compared using the Chi-square test. Continuous variables are expressed as mean ± SD for normally distributed data and median and interquartile range for non-normally distributed data. These variables were tested for normality using the Komorolgov-Smirnov test. The data with normal distribution were compared using the Student’s *t*-test, and the Mann–Whitney U test was used to compare the data with non-normal distribution. The receiver operating characteristics (ROC) curve was analyzed, and the area under the curve (AUROC) was calculated to establish diagnostic accuracy. Correlations were evaluated with Spearman’s correlation index. The factors associated with early diagnosis of infections in patients with LC and overt HE were identified using univariate and multivariate logistic regression. In this study, *p* < 0.05 was set as the significance level.

## 3. Results

### 3.1. Patients Characteristics

**Figure 1:**
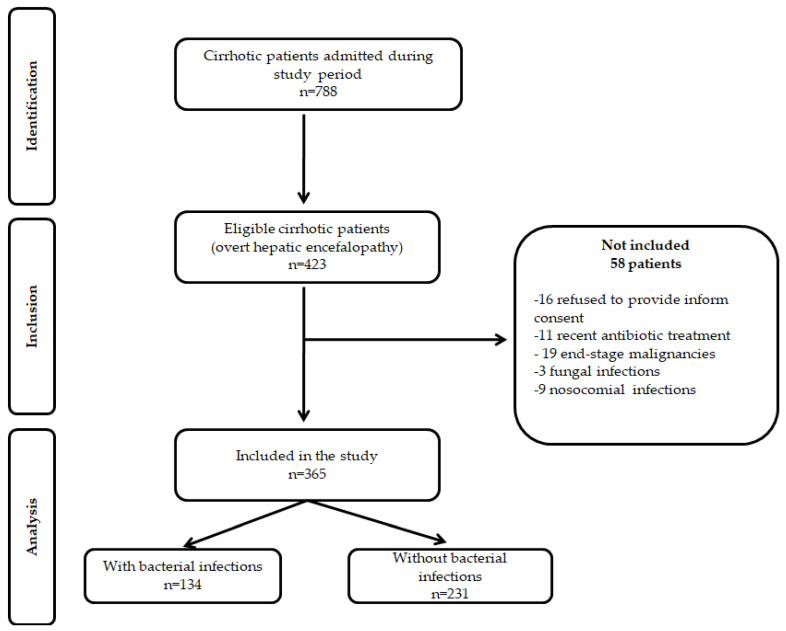
STROBE flow diagram of patients included and excluded from the study.

During the study period, 788 cirrhotic patients were admitted to our department, 423 were eligible for the study, and 365 patients fulfilled the inclusion criteria (Figure 1). There were no differences regarding gender, age, LC etiology, or severity between patients included in the study compared to those excluded.

The 365 patients included in the study had a median age of 59 years, 61.9% of whom were male. The main etiology of LC was an alcoholic (84.7%), and 25 patients were diagnosed with acute alcoholic hepatitis (10.8%). Table 1 presents the characteristics of the participants. Most of the patients had comorbidities (64.7%). In the study group, the majority of the patients had ascites (89.3%), and 23.0% of the patients had variceal bleeding. In our cohort, 75 patients (20.5%) were diagnosed with acute kidney injury. The presepsin levels were not significantly different between patients with or without acute kidney injury (961 pg/mL vs. 1013 pg/mL, *p* = 0.754). More than half of the patients included in this study had grade 2 HE (58.4%), with a median presepsin level of 1013 pg/mL.

### 3.2. Infectious Complications

Infections were diagnosed in 134 patients (36.7%), of whom 11 patients (8.2%) had more than two infections. Most patients had community-acquired infections (76.1%). There was no difference in the presence of alcoholic hepatitis in patients with or without infections (10.4% vs. 10.8%, *p* = 0.553).

The most frequent site of infection was UTI (31.3%), followed by SBP (25.4%) and CDI (17.9%), and the Gram-negative bacteria were most frequently identified as etiologic agents (56.6%) (Table 2). Nine patients had community-acquired CDI, and 15 patients health-care associated CDI. Sepsis was diagnosed in 42 patients (31.3%) and was secondary to SBP, pneumonia, or UTI. Patients with sepsis had a median presepsin of 5032 pg/mL, significantly higher than those without sepsis- 2588 pg/mL, *p* = 0.002. Twelve patients (28.6%) were diagnosed with septic shock, with a median presepsin of 4317 pg/mL and no significant difference compared to presepsin levels in sepsis patients (*p* = 0.576).

There was no significant difference among the three study groups in terms of age, LC etiology, and comorbidities (Table 1). However, patients with infections had a more severe LC, as MELD score (*p* = 0.002) and Child-Pugh class (*p* = 0.033) revealed. The cirrhotic patients diagnosed with infections had higher levels of CRP, although we found no differences regarding white blood cells and NLR levels. In the study group, 43% of the patients had chronic non-selective beta-blockers (NSBBs) treatment, and 15.1% had long-term proton pump inhibitors. There was a statistically significant difference between the patients with and without NSBB treatment in terms of infection development (31.3% vs. 49.7%, *p* = 0.001). More than half of the patients had previous HE episodes (198 patients, 54.2%), and 96 patients (26.3%) received rifaximin as the HE secondary prophylaxis. There was no difference among the study groups regarding previous rifaximin treatment (26.1% vs. 26.4%, *p* = 0.901).

### 3.3. Presepsin and Infectious Complications

The presepsin level was higher in patients with infections than those without infections (3167 vs. 500, *p* < 0.001). The presepsin levels were higher in patients with Gram-negative infections than in those with Gram-positive infections (5346 pg/mL vs. 2568 pg/mL, *p* < 0.001).

**Figure 2:**
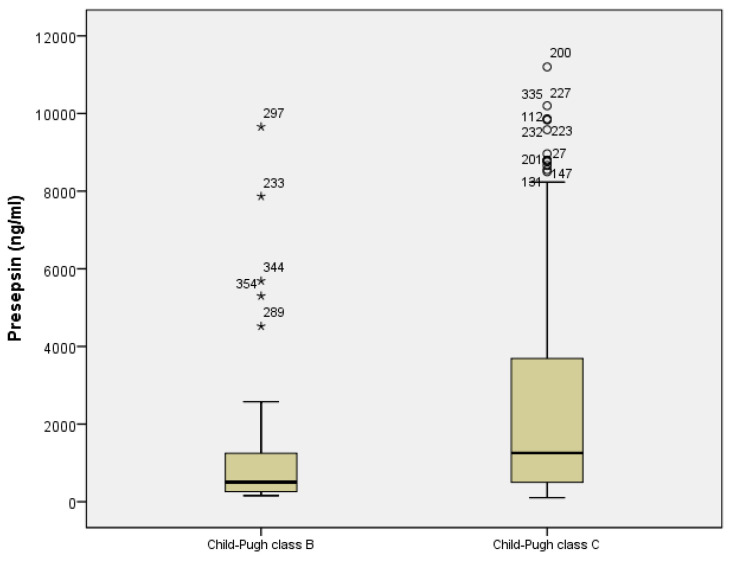
Relationship between presepsin level and liver cirrhosis severity.

Moreover, the presepsin level increased with LC severity in the Child-Pugh class (505 pg/mL vs. 1256 pg/mL, *p* = 0.017) (Figure 2), and there was a positive correlation between the presepsin level and the MELD score (r = 0.130, *p* = 0.046).

**Figure 3:**
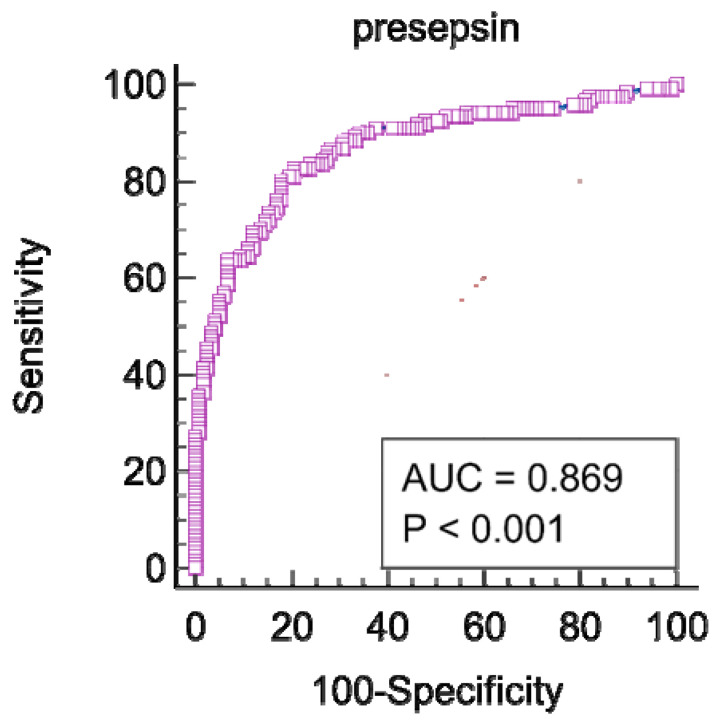
ROC curve for presepsin in predicting infections.

The ROC analysis results demonstrated that the best cut-off value for presepsin for infections detection was 980 pg/mL with a sensitivity of 80.17%, specificity of 82.5% (AUROC 0.869, CI 95%: 0.819–0.909, *p* < 0.001, Youden index J of 0.622), a positive predictive value of 40.63%, and a negative predictive value of 96.53% (Figure 3). The optimal cut-off value of the CRP, white blood cells and NLR levels to discriminate between cirrhotic patients with or without bacterial infections are presented in Table 3.

Multivariate logistic regression analysis demonstrated that the presepsin level higher than 980 pg/mL represents a factor associated with the diagnosis of infections in patients with LC and overt HE, along with the CRP value of more than 2.81 mg/dL (Table 4).

During hospitalization, 103 patients (28.2%) died. The mortality rate was higher in patients with LC and infections than in those without infections (35.1% vs. 24.2%, *p* = 0.027). The patients who died had higher baseline presepsin levels than survivors (2135 pg/mL vs. 780 pg/mL, *p* = 0.044).

## 4. Discussion

Bacterial infections complicate the course of LC and determine an increased rate of decompensation, acute-on-chronic liver failure, and mortality [23]. The early diagnosis and prompt treatment of infections prevent LC from further decompensation and sepsis development and may decrease mortality rates [5].

An increased intestinal permeability characterizes liver cirrhosis due to portal hypertension. This determines a high level of LPS in cirrhotic patients’ blood without evidence of bacterial infections [24]. Presepsin is the soluble part of CD14, a co-receptor for lipopolysaccharides ligands having higher levels in the cirrhotic population without infections than in the general population [19]. For this reason, different presepsin cut-offs should be identified in patients with LC [10].

The present study’s findings demonstrated that the serum presepsin level >980 pg/mL had the most appropriate specificity and sensitivity to identify bacterial infections in patients admitted with LC and overt HE. Moreover, the presepsin levels were directly correlated with LC severity assessed by the MELD score and the Child-Pugh class.

Prospective studies, including patients with compensated and decompensated LC, demonstrated that a presepsin level of >600 pg/mL was associated with an increased one-year liver-related mortality [11]. They also documented that cirrhotic patients have higher levels of presepsin even at the compensated stage and without infection complications than presepsin levels previously reported in the general population. This fact could reflect spontaneous bacterial translocation associated with LC [25]. In the general population, the presepsisn level in adult subjects was 55–184 pg/mL, whereas our study’s median presepsin level in patients without infections was 500 pg/mL. This is in accordance with previous data that demonstrated a higher presepsin level in patients with LC without bacterial infections due to persistent intestinal bacterial translocation [11,26].

Papp et al. concluded that presepsin had better diagnostic accuracy in patients with LC and infections-associated organ failure. The association between CRP and presepsin increases the diagnostic accuracy of these biomarkers in terms of infection diagnosis in patients with LC [19]. They also demonstrated that the presepsin cut-off for infection diagnosis was 844 pg/mL, above the value previously reported for the general population (400–600 pg/mL) [25,27]. Furthermore, Novelli et al. showed that presepsin could discriminate bacterial infections from other types of infection with different cut-off values according to the type of infection [13].

In a meta-analysis of 10 studies, a high presepsin level in the first 24 h after admission was a predictor of mortality [28]. These data were also confirmed in the present study.

The relatively low positive predictive value could sustain the necessity of combined serum biomarkers for early diagnosis of infections in patients with liver cirrhosis and overt HE. Considering these data, we analyzed the role of CRP, white blood cells, and NLR in the early diagnosis of infections in patients with LC and overt HE. We found that CRP level was higher in patients with infections, although no differences were demonstrated for white blood cells or NLR levels between the two study groups. The severity of LC could explain this; most of the patients are classified as Child-Pugh C, associated with significant portal hypertension and hypersplenism. In clinical practice, the diagnosis of infection should be set up in the context of characteristic symptoms and laboratory tests, including the presepsin level. Samples collections for culture should be obtained in each cirrhosis patient admitted with overt HE. However, the empirical antibiotic treatment could be considered if the presepsin level was >860 pg/mL and CRP levels were more than 2.81 mg/dL. The goal is to prevent the evolution of sepsis and septic shock, considering that this diagnosis is challenging in patients with HE because they already have an altered mental state because HE, leucopenia, arterial hypotension, or hypoxemia, secondary portal hypertension, and all the sepsis criteria include all these items.

In our study, the cirrhotic patients with infections received less frequent NSBBs than patients without infections due to the severity of the disease and NSBBs intolerance or contraindications, and NSBBs are not risk factors for developing infections in patients with LC and overt HE.

The present study has some strengths and limitations. This is the first prospective cohort study examining the role of presepsin in the early diagnosis of infections in patients with LC and overt HE. The limitations of our research consist of the small number of patients and short follow-up period. Moreover, above 80% of the study population had the alcoholic etiology of LC, and our findings could not be transferred to patients with other LC etiologies. Since the data are not homogeneous, different cut-offs should be defined for diagnosing infections in patients with non-alcoholic LC and overt HE or other LC complications.

## 5. Conclusions

Presepsin may be an adequate non-invasive tool for the early diagnosis of infections in patients with LC and overt HE to decrease mortality rates during hospitalization. In this regard, further prospective large cohorts study should confirm the present findings.

## Figures and Tables

**Table 1:** Clinical and laboratory characteristics of the study groups.

Parameter	All Patients *n* = 365	With Bacterial Infections *n* = 134	Without Bacterial Infections *n* = 231	*p*
Age, years, median (IQR)	59 (16)	56.5 (20)	60 (15)	0.397
Male sex, *n* (%)	226 (61.9)	73 (54.5)	153 (66.2)	**0.026**
Comorbidities, *n* (%)	236 (64.7)	87 (64.9)	149 (64.5)	0.935
Cirrhosis etiology, *n* (%)				0.052
Alcoholic	309 (84.7)	107 (79.8)	202 (87.4)	
Non-alcoholic	56 (15.3)	27 (20.2)	29 (12.5)	
Child-Pugh class, *n* (%)				**0.033**
B	67 (18.4)	17 (12.7)	50 (21.6)	
C	298 (81.6)	117 (87.3)	181 (78.4)	
MELD score, median (IQR)	21 (11)	23 (12)	20 (10)	**0.002**
Total bilirubin (mg/dL), median (IQR)	3.88 (8.91)	4.9 (12.8)	4.8 (12.5)	0.119
INR, median (IQR)	1.9 (0.83)	1.99 (0.99)	1.86 (0.8)	0.251
Creatinine (mg/dL), median (IQR)	1.17 (1.35)	1.45 (1.34)	1.07 (1.27)	**0.025**
HE grade 2/3/4, *n* (%)	213/102/50 (58.4/27.9/13.7)	69/46/19 (51.5/34.3/14.2)	144/56/31 (62.3/24.2/13.5)	0.089
Serum Na (mmol/L), median (IQR)	134 (9)	135 (9)	134 (11)	0.641
C-reactive protein, (mg/dL), median (IQR)	2.74 (4.43)	4.17 (5.37)	2.13 (3.76)	**<0.001**
White blood cell count (×10^9^/L), median (IQR)	4.5 (4)	4.7 (3.9)	4.5 (4)	0.149
NLR, median (IQR)	3.5 (6.7)	4.2 (7.3)	3.2 (4.5)	0.055
Presepsin, (pg/mL), median (IQR)	1013 (3160)	3167 (4392)	500 (504)	**<0.001**
NSBB, *n* (%)	157 (43)	42 (31.3)	115 (49.7)	**0.001**
PPIs, *n* (%)	55 (15.1)	14 (10.4)	39 (16.8)	0.129
Rifaximin, *n* (%)	96 (26.3)	35 (26.1)	61 (26.4)	0.901
In-hospital mortality, *n* (%)	103 (28.2)	47 (35.1)	56 (24.2)	**0.027**

IQR, interquartile range; MELD, Model of End-Stage Liver Disease; INR, International Normalized Ratio; HE, hepatic encephalopathy; NLR, neutrophil-to-lymphocyte ratio; NSBB, non-selective beta-blockers; PPIs, proton pump inhibitors.

**Table 2:** Bacteria identified in the study groups.

Pathogen	Urinary Tract 43 (31.3%)	SBP 34 (25.4%)	Respiratory Tract 13 (9.7%)	CDI 24 (17.9%)	Bloodstream 16 (11.9%)	Skin 5 (3.7%)
*Escherichia coli*	29	7	-	-	7	-
*Klebsiella pneumoniae*	2	1	2	-	2	2
*Enterococcus faecium*	7	2	1	-	2	-
*Pseudomonas aeruginosa*	1	-	-	-	-	-
*Staphilococcus aureus*	-	-	1	-	2	2
*Streptococcus pyogenes*	-	-	-	-	3	-
*Morganella morganii*	-	-	-	-	-	1
*Moraxella catharralis*	-	-	1	-	-	-
*Clostridioides difficile*	-	-	-	24	-	-

SBP, spontaneous bacterial peritonitis; CDI, *Clostridioides difficile* infection.

**Table 3:** Comparisons of discriminating abilities of tested biomarkers presented as areas under the curve (95% CI).

Tested Biomarker	AUROC (95% CI)	Cut-off Value	Sensitivity, (%)	Specificity, (%)	*p*-Value
Presepsin	0.869	980 pg/mL	80.17	82.5	**<0.001**
CRP	0.673	2.81 mg/dL	67.7	62.6	**<0.001**
White blood cells	0.542	6.40 × 10^9^/L	30.4	73.4	0.181
NLR	0.566	3.2	59.3	50.0	**0.034**

AUROC, area under the receiver operating; CRP, C reactive protein; NLR, neutrophil-to-lymphocyte ratio.

**Table 4:** Univariate and multivariate logistic regression analyses of factors associated with infections in patients with LC and overt HE.

Parameter	Univariate Analysis			Multivariate Analysis		
	OR	CI 95%	*p*-Value	OR	CI 95%	*p*-Value
Male gender	0.81	0.679–0.974	**0.024**	0.86	0.415–1.822	0.710
Presepsin ≥ 980 pg/mL	4.39	2.960–6.524	**<0.001**	9.41	6.880–16.293	**<0.001**
CRP ≥ 2.81 mg/dL	1.65	1.347–2.028	**<0.001**	2.94	1.367–6.344	**0.006**
WBC > 6400 × 10^9^/L	1.04	0.785–1.517	0.605	1.75	0.755–4.072	0.192
NLR ≥ 3.2	1.25	1.046–1.499	**0.023**	1.38	0.655–2.949	0.392
MELD ≥ 18	1.20	1.062–1.362	**0.008**	1.14	0.453–2.904	0.773
Child-Pugh class C	1.16	1.063–1.276	**0.004**	0.91	0.292–2.847	0.873
Previous NSBBs treatment	0.64	0.486–0.849	**0.001**	0.67	0.305–1.514	0.344
Previous rifaximin treatment	0.97	0.684–1.397	0.901	0.82	0.320–2.110	0.982
Previous PPIs treatment	0.65	0.376–1.143	0.172	1.34	0.455–3.989	0.590

CRP, C-reactive protein; WBC, white blood cells; MELD, Model of End-Stage Liver Disease; NLR, neutrophil-to-lymphocyte ratio; NSBBs, non-selective beta-blockers; PPIs, proton pump inhibitors.

## Data Availability

The data presented in this study are available on request from the corresponding author. The data are not publicly available because they are property of the Institute of Gastroenterology and Hepatology, Iasi, Romania.
